# Prognostic role of pretreatment albumin-to-alkaline phosphatase ratio in locally advanced laryngeal and hypopharyngeal cancer: Retrospective cohort study

**DOI:** 10.7150/jca.61445

**Published:** 2021-08-27

**Authors:** Jialing Wu, Kaiyun You, Yanhui Jiang, Ting Shen, Juanjuan Song, Changlong Chen, Yimin Liu

**Affiliations:** Department of Radiation Oncology, Sun Yat-Sen Memorial Hospital, Sun Yat-Sen University, Guangzhou, China.

**Keywords:** Albumin-to-alkaline phosphatase ratio, Larynx, Hypopharynx, Prognosis, Surgery, Head and neck squamous cell carcinoma

## Abstract

**Background:** This study was designed to assess the prognostic significance of pretreatment albumin-to-alkaline phosphatase ratio (AAPR) in locally advanced laryngeal and hypopharyngeal cancer (LA-LHC).

**Materials and Methods:** The clinical data of 341 patients with locally advanced laryngeal and hypopharyngeal cancer diagnosed between March 2007 and December 2018 were retrospectively collected and analyzed. The optimal cut-off value of AAPR for evaluating DFS was determined using the ROC curve, and 0.4912 was selected. Based on pretreatment AAPR values, patients were divided into two groups (low vs. high AAPR). Survival analysis was used to investigate the survival distribution between the groups. Univariate and multivariate analyses were performed to evaluate the prognostic value of AAPR. Based on the results of the multivariate analysis, we further developed models of DFS and OS. We assigned low AAPR, N1-3, age ≥65 years, and positive vascular invasion one score, respectively.

**Results:** Survival analysis demonstrated that the survival of patients with low and high AAPR was significantly different (low vs. high AAPR: 5-year DFS, 46.0 vs. 71.9%, p<0.001; 5-year OS, 69.0 vs. 72.6%, p<0.001). Univariate and multivariate analyses further showed that pretreatment AAPR served as an independent indicator in LA-LHC. Moreover, survival analysis showed that patients with high model score had poorer DFS and OS (5-year DFS: 58.1, 42.7, 26.9 and 9.1% of score zero, one, two, and three respectively, p<0.001; 5-year OS: 63.0, 50.3, 34.1 and 28.6% of score zero, one, two, and three respectively, p<0.001).

**Conclusion:** Pretreatment AAPR could be an independent prognostic indicator in patients with LA-LHC. Incorporating AAPR into the risk stratification model might better categorize patients with worse oncological outcomes and support treatment strategy making.

## Introduction

Of the 8.78 million cases of head and neck cancer (HNC) worldwide in 2020, with 4.44 million deaths, approximately 30% are attributed to laryngeal and hypopharyngeal cancer 1. Among HNC, laryngeal and hypopharyngeal cancer (LHC), which pertains to the upper aerodigestive tract, is confronting the same carcinogenic substances, such as alcohol, nicotine, nitroso compounds, and HPV 2, 3. Patients with LHC possess akin treatment options and similar clinical management strategies 4, 5. More than 50% of LHC patients are diagnosed with the locally advanced disease during their initial clinical consultation 6. The 5-year overall survival (OS) and disease-free survival (DFS) of locally advanced laryngeal and hypopharyngeal cancer (LA-LHC) were in the range of 47.7-60.2% and 42.2-69.4%, respectively, according to various publications 7-12.

Treatment options for patients with LHC include radical surgery and organ-preserving treatment modalities. Over the last few decades, radiotherapy and chemotherapy have gradually achieved a dominant position 12-15. The importance of radical surgery remains, especially for patients with locally advanced stage situations, as surgery has a preferable survival rate 16. The main surgical strategies prescribed to patients with LHC are total (or partial) laryngectomy and laryngopharyngectomy combined with cervical lymph node dissection 5. Postoperative adjuvant therapy is administered to patients with adverse clinicopathologic features 17. Considering the unsatisfactory oncologic outcomes of LA-LHC, the development of novel prognostic factors that can predict clinical consequences is warranted.

Patients with LA-LHC usually suffered either from tumors of the T3-4 stage or from lymph node metastasis of the N2-3 stage. Primary tumors categorized as T3-4 are larger than 4 cm in the greatest dimension or extensive invasion of surrounding tissues. Lymph node statuses categorized as N2-3 are larger than 3 cm, multiple in number, or extranodal extension 18. Both conditions resulted in an increased possibility of infection and progressive dysphagia, ultimately leading to cachexia and poor clinical outcomes. In this study, we aimed to evaluate and identify prognostic factors of the oncological outcomes of LA-LHC.

Serum albumin (ALB) is an indicator of nutritional status, liver function, renal function, and immunological function. Serum alkaline phosphatase (ALP) projects systemic inflammation and liver function. Both ALB and ALP levels were significantly associated with the prognosis of cancer patients. Thus, we hypothesized that combining ALB with ALP into one factor could better indicate the survival outcomes of patients with LA-LHC. The prognostic value of the albumin-to-alkaline phosphatase ratio (AAPR) was first reported in patients with hepatocellular carcinoma in 2015 19. This novel prognostic factor has been recently exploited in several malignancies (e.g., nasopharyngeal carcinoma, liver cancer, renal cell carcinoma, pancreatic ductal adenocarcinoma, etc.,) and low AAPR was associated with worse survival outcomes 20-24. Although research on using AAPR for various malignances has been conducted, studies on the prognostic value of AAPR for LA-LHC are still scarce. Here, we performed a retrospective study on the association between AAPR and LA-LHC prognosis.

## Materials and methods

### Patients

Clinical data of 341 laryngeal or hypopharyngeal cancer patients diagnosed between March 2007 and December 2018 at our institution were retrospectively reviewed and collected. We included patients: (1) with eligible pretreatment serum ALB and ALP, (2) with histologically confirmed laryngeal or hypopharyngeal cancer, (3) with stage III or IV disease, and (4) older than 18 years. We excluded patients with: (1) lack of available pretreatment ALB or ALP, (2) distant metastasis, (3) stage I or II disease. A total of 341 patients were included in this study. Clinicopathological records, including age, sex, margin status, vascular invasion, surgery, adjuvant radiotherapy, adjuvant chemotherapy, TNM stage, and laboratory test results, including ALB and ALP, were collected and evaluated.

### Albumin-to-alkaline phosphatase ratio (AAPR)

Patients' peripheral blood samples were extracted for laboratory examination within 2-4 weeks before the initiation of any therapy. Normal serum ALB levels were determined to 35.0-50.0 g/L. Normal serum ALP levels were determined to 45 - 125 U/L for male and 50 -135 U/L for female. Pretreatment AAPR was calculated by dividing ALB by ALP. The optimal cut-off value of AAPR evaluating DFS was worked out by employing ROC curve, and 0.4912 was selected as it had the maximum Youden index value (sensitivity: 56.7%; specificity: 68.7%; Youden index: 0.254). Based on pretreatment AAPR levels, patients were classified into high AAPR group (AAPR ≥ 0.4912) and low AAPR group (AAPR < 0.4912).

### Clinical staging

Before anti-tumor treatments, all patients underwent clinical assessments, including physical inspections, laboratory tests, chest radiography, electronic laryngoscopy and esophageal barium meal examination. Contrast-enhanced magnetic resonance imaging (MRI) and computed tomography (CT) of the larynx and hypopharynx used to examine the tumor size, surrounding invasion, and lymph node metastasis status were performed in selected patients. Abdominal ultrasonography and whole-body bone scan using single-photon emission computed tomography (SPECT), which aimed to detect distant metastasis, were performed in some patients.

Tumor-node-metastasis (TNM) classification was based on pretreatment examinations and postoperative pathological findings determined by the 7th edition of the American Joint Committee on Cancer (AJCC) for laryngeal and hypopharyngeal cancer.

### Treatment and follow up

The treatment plan was discussed and determined by experienced otolaryngologist, oncologist, and radiologist. All patients received surgery-based combined therapy. Surgery modalities included larynx and hypopharynx tumor excision without cervical lymph node dissection and total (or partial) laryngectomy or laryngopharyngectomy, with or without cervical dissection.

Postoperative therapy was accomplished in patients with adverse pathologic features such as extranodal extension, positive surgery margin, pT3 or pT4, multiple positive lymph nodes, vascular invasion, etc. 153(44.3%) patients were allocated adjuvant chemotherapy. In this group of patients, the chemotherapy scheme of TP (taxol and platinum) (84.9%) was predominantly used, in addition to other platinum-based chemotherapy. Adjuvant radiotherapy (3D conformal RT or IMRT) was prescribed to some patients. The total doses of the tumor bed and lymphatic drainage area were 60-66Gy and 50-56Gy, respectively (1.8-2.0Gy/fraction; 5 times per week; in 6-7 weeks).

Patients were monitored every 3 months for the first 2 years, every 6 months for the subsequent 3-year, and annually thereafter.

### Statistical analysis

Data statistical analyses were conducted by using SPSS 25.0 software, and two-sided P-value ≤ 0.05 was considered statistically significant. Chi-square test was performed to evaluate the relevance between categorical variables and AAPR. Survival curves were worked out by Kaplan-Meier method, and the significant differences of survival between groups were calculated by log-rank test. Univariate and multivariate analyses were performed to further chase down the risk factors of DFS and OS.

## Results

### Patients' baseline characteristics

Baseline clinical data of 341 patients with locally advanced laryngeal and hypopharyngeal cancer (LA-LHC) were retrospectively collected and analyzed. All patients enrolled in this study were East Asian. The median follow-up time was 4.67 years (range: 0.42-13.25 year). All patients were classified into two groups: low AAPR group (≤ 0.4912) and high AAPR group (>0.4912). The study included 331 males and 10 females. The median age was 59 years (range, 29-95 years). A total of 284 (83.3%) patients were heavy cigarette smokers, and 160 (46.9%) patients had a history of heavy drinking. In terms of the 7th edition TNM staging system of AJCC, all patients were presenting at stage III-IV. Fifty-five (16.1%) patients were defined as T1-2 stage, and 286 (83.9%) patients were classified as T3-4 stage. Concerning lymph node metastasis, the numbers of patients with N0 and N1-3 were 124 (36.4%) and 217 (63.6%) respectively. The mean values of ALB and ALP were 40.1 (g/L, interquartile range: 37.8-43.0 g/L) and 78.84 (U/L, interquartile range: 66-95 U/L). At the last follow-up, 128 patients died, and 213 patients were alive. Recurrence was observed in 126 patients. The patients' clinical and pathological information is summarized in **Table 1**.

### Survival analysis

**Figure 1:**
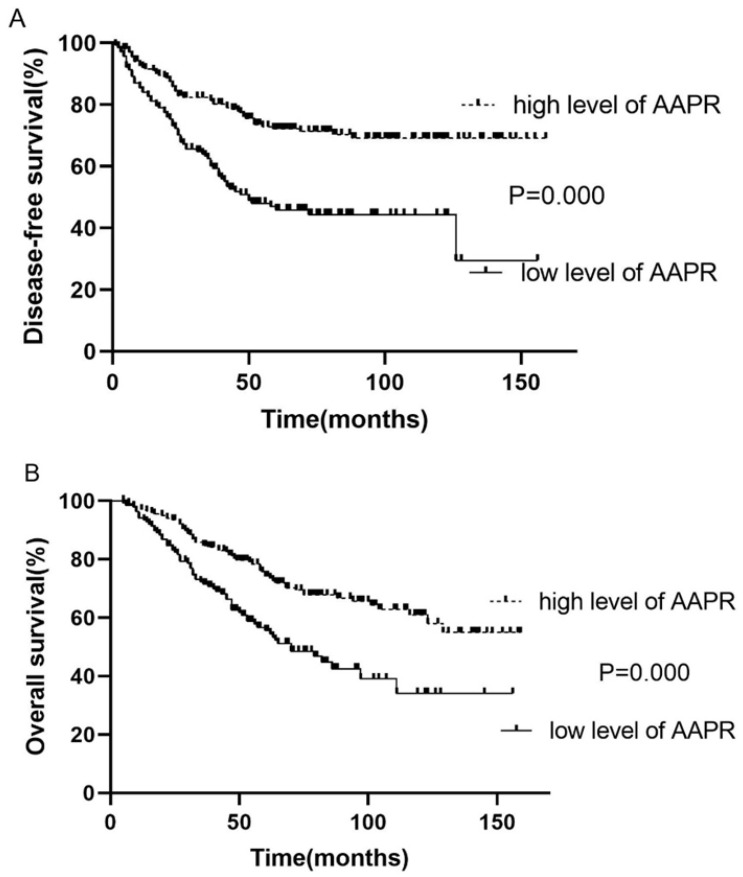
Kaplan-Meier curve for disease-free survival and overall survival, stratified by pretreatment AAPR, cutoff value: 0.4912.

Kaplan-Meier survival analysis and log-rank test demonstrated that the survival of patients with low and high AAPR was significantly different (low vs. high AAPR: DFS, 3-year, 58.6 vs. 81.2%, 5-year, 46.0 vs. 71.9%, p<0.001; OS, 3-year, 70.8 vs. 84.6%, 5-year, 69.0 vs. 72.6%, p<0.001). Moreover, survival analysis showed that patients with low AAPR levels were significantly correlated with inferior LRFS (low vs. high AAPR, 3-year, 63.4 vs. 85.2%, 5-year, 55.7 vs. 78.6%, p<0.001). However, the relationship between AAPR and DMFS was not significant (p=0.140) (**Table 2**). The Kaplan-Meier curves of DFS and OS of patients with AAPR ≤0.4912 and AAPR >0.4912 are shown in **Fig. 1**.

### Univariate analysis

Univariate analysis revealed that low pretreatment AAPR was an unfavorable determinant of DFS (low vs. high AAPR, p<0.001, HR=2.370, 95% CI 1.667-3.378) and OS (low vs. high AAPR, p<0.001, HR=2.049, 95% CI 1.443-2.901). Additionally, N status was significantly associated with both DFS and OS. Vascular invasion was associated with DFS, whereas age was correlated with OS. However, sex, T stage, margin status, adjuvant therapy, smoking history, and drinking history were not associated with survival consequences (**Table 3**).

### Multivariate analysis

To identify the independent risk factors for DFS and OS, we further performed multivariate analysis. Multivariate analysis further confirmed that AAPR (low vs. high AAPR, DFS, p<0.001, HR=2.457, 95% CI 1.718-3.509; OS, p<0.001, HR=2.016, 95% CI 1.418-2.865), N status (N0 vs. N1-3, DFS, p=0.024, HR=0.637, 95% CI 0.431-0.942; OS, p=0.018, HR=0.632, 95% CI 0.432-0.925) were independent prognostic factors for both DFS and OS. Besides, vascular invasion (yes vs. no, p=0.002, HR=2.193, 95% CI 1.348-3.571) and age (<65 vs. ≥65, p=0.009, HR=0.607, 95% CI 0.418-0.881) were independent risk factors for DFS and OS, respectively (**Table 4**).

### Prognostic models

**Figure 2:**
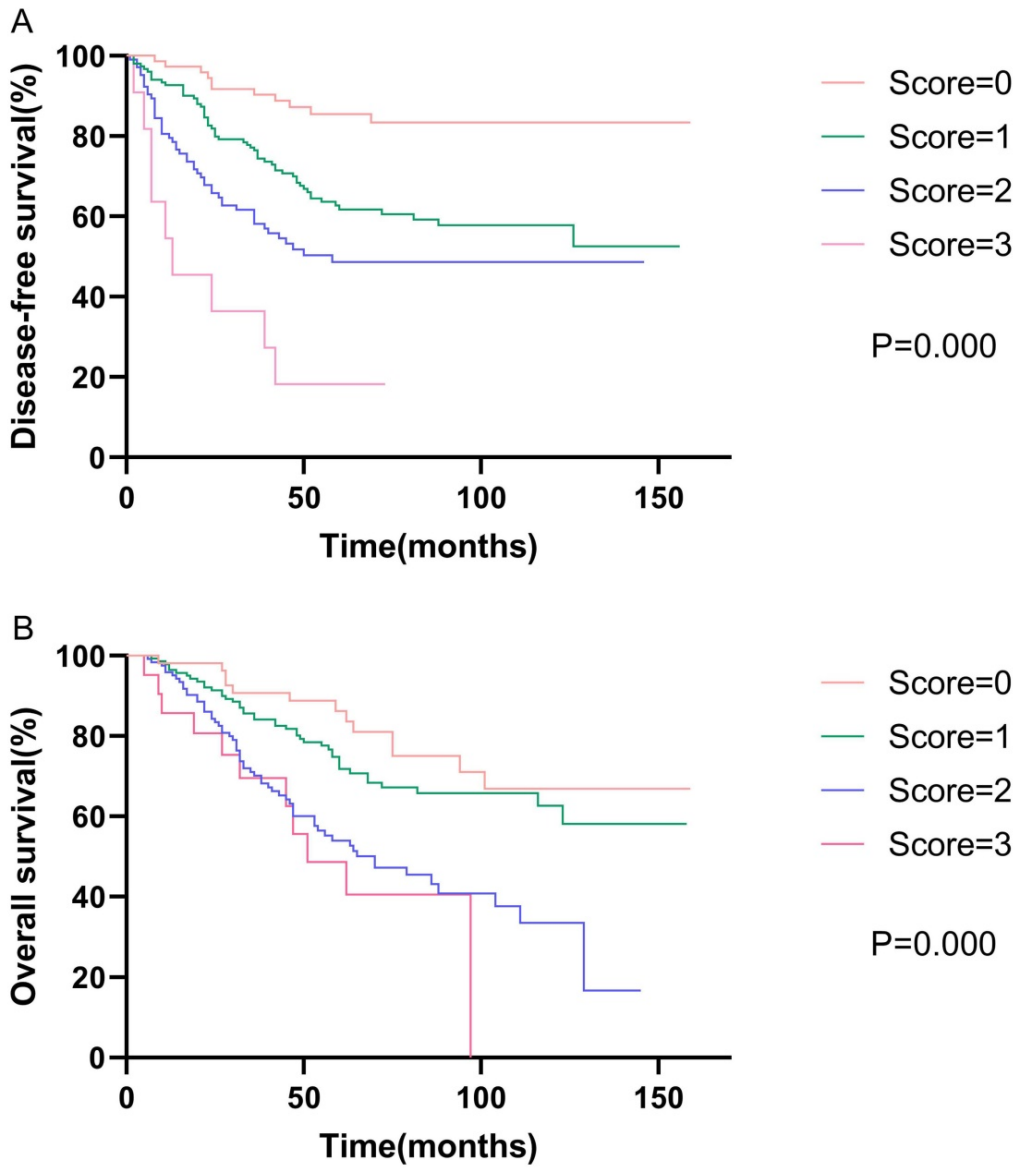
Kaplan-Meier curve for disease-free survival and overall survival, stratified by prognostic models of DFS and OS.

Based on the results of the multivariate analysis, we further developed models of DFS and OS. The DFS model consisted of AAPR, N status, and vascular invasion, while the OS model consisted of AAPR, N status, and age. We assigned low AAPR, N1-3, old age (≥65 years), and positive vascular invasion, one score respectively. Survival analysis showed that patients with high model scores had poorer DFS and OS. The 5-year disease-free survival rates were 58.1%, 42.7%, 26.9%, and 9.1% for scores of zero, one, two, and three, respectively (p<0.001). The 5-year overall survival rates were 63.0%, 50.3%, 34.1%, and 28.6% for scores of zero, one, two, and three, respectively (p<0.001). The Kaplan-Meier curves of prognostic models of DFS and OS are presented in **Fig. 2**.

## Discussion

Laryngeal and hypopharyngeal cancers have frequently been investigated as a single entity and shared similar treatment strategies 4, 7, 8, 10, 11, 14. To the best of our knowledge, this is the first study to investigate the prognostic value of pretreatment AAPR in locally advanced laryngeal and hypopharyngeal cancer (LA-LHC). In this study, we found that low AAPR was associated with less favorable survival outcomes, and pretreatment AAPR served as an independent indicator in LA-LHC.

AAPR is derived from serum ALB and ALP, which can be easily and reproducibly accessed and extracted from blood tests. The relatively low levels of ALB or hypoalbuminemia in patients with cancer can be explained by: (1) stress response provoked by illnesses, (2) low protein intake, and (3) intercurrent infections 25. Researchers have found a correlation between low serum ALB level and immunological dysfunction 26. The resulting diminished immune function fuel the growth of tumors and further jeopardize the efficacy of anti-cancer treatment. One study examined the clinical utility of pretreatment serum ALB in patients with head and neck squamous cell carcinoma (HNSCC). The results showed that there was a relationship between low pretreatment albumin and increased wound infection and inferior OS.

Moreover, this relationship was most prominent in patients with upper aerodigestive squamous cell carcinoma 27. Parallel to what had been observed, Lim et al. also reported that low pretreatment serum ALB significantly increased the risk of tumor progression and cancer-related death 28. Another study investigated the prognostic role of serum ALB in patients with advanced HNSCC who underwent surgery and flap reconstruction and determined that preoperative hypoalbuminemia was a poor prognostic indicator in this population 29. Also, studies have shown that low serum ALB is an unfavorable determinant in other malignancies, including oral, breast, and vulvar cancers 30-32.

Alkaline phosphatase (ALP) is a serum metalloenzyme. The elevation of ALP projects the presence of hepatobiliary disease and liver and bone metastases. ALP is an indicator of hepatobiliary tract obstruction^33^ and elevated osteoblastic activity^34^, and thus serving as a very subtle predictor of liver and bone metastases in patients with cancer. The prognostic role of ALP has been observed in many studies, which have consistently revealed that ALP is an unfavorable factor, as well as an indicator of cancer recurrence and distant metastasis 35-37. Specifically, study disclosed that high pretreatment ALP was significantly correlated with poor survival in patients with laryngeal cancer 38.

Overall, pretreatment AAPR reflected primary tumor load, inflammatory condition, and nutrition condition. AAPR appeared to perform better than ALP and ALB alone in predicting the survival outcomes of cancer patients. A meta-analysis conducted by Tian et al. evaluated the possible utility of AAPR in solid cancers, and showed that low pretreatment AAPR was associated with worse OS in solid cancers, such as lung cancer, hepatocellular carcinoma, pancreatic ductal adenocarcinoma, and nasopharyngeal carcinoma 23. Burgeoning evidences suggested the important prognostic value of AAPR 20, 21, 39, 40, which were consistent with our findings.

Additionally, our findings revealed that N status was an independent prognostic factor for both DFS and OS, consistent with previous reports 41, 42. Vascular invasion and age were independent risk factors for DFS and OS, respectively. Vascular invasion is associated with lymph node metastasis 43, 44 and a well-established survival predictor in various cancers, including esophageal, upper urinary tract urothelial, oral tongue squamous cell, and adenoid cystic carcinoma 43-46. In our study, we chose 65 years as the inflection point; however, Abrahão et al. selected 71 years and reported that patients ≥ 71 years were associated with increased mortality risk 47. Our study showed that heavy alcohol consumption and tobacco use were prevalent among head and neck cancer patients. Studies have demonstrated that tobacco smoking is associated with mortality in head and neck cancers 48, 49. However, our study failed to support this relationship, and we speculated that the retrospective nature and relatively small size of our study account for the negative result. Nevertheless, our finding of alcohol consumption was consistent with previous literature 48.

More intriguingly, we constructed models that incorporated AAPR, N status, and vascular invasion for DFS and contained AAPR, N status, and age for OS. Survival analysis revealed that these models improved the stratification of patients with various risks. Incorporating these models into clinical use may help clinicians in treatment making.

Some limitations of this study should be noted. First, the retrospective nature of this study inevitably dampened its utility. Second, AAPR is based on ALB and ALP. Since abnormal ALB and ALP can result from various non-cancer situations, and thus AAPR is not a cancer-specific biomarker. Both ALB and ALP abnormalities can affect AAPR. The cause of abnormal ALB and ALP include malnutrition, benign liver disease, and renal dysfunction, among others. Exploiting this intriguing prognostic factor requires further diligence and robust studies. Third, our study focused on patients who received surgery-based treatment, which was the main treatment option for patients with LA-LHC in our institution; thus, our results might fail to extend to patients who underwent definitive chemoradiotherapy. In addition, external validation of our main findings and conclusions is required. Moreover, since the patients collected and evaluated in this study were predominantly males, the results did not extrapolate well to female patients with LA-LHC.

## Conclusion

For the first time, our study sheds new light on the prognostic impact of AAPR in patients with LA-LHC. Our results showed that pretreatment AAPR was an independent prognostic factor in patients with LA-LHC. Incorporating AAPR into stratification models may better forecast the oncological consequences of patients with LA-LHC. More well-designed, prospective and multi-center studies are required to further verify the clinical value of AAPR.

## Figures and Tables

**Table 1:** Associations between AAPR and clinicopathological characteristics

Variable	Low level of AAPR (N=139)	High level of AAPR (N=202)	*P* value
**Age, year**			0.482
<65	97 (69.8%)	148 (73.3%)	
≥65	42 (30.2%)	54 (26.7%)	
**Sex**			0.482
male	136 (97.8%)	195 (96.5%)	
female	3 (2.2%)	7 (3.5%)	
**T stage^†^**			0.469
T1-2	20 (14.4%)	35 (17.3%)	
T3-4	119 (85.6%)	167 (82.7%)	
**N status^†^**			0.204
N0	45 (32.4%)	79 (39.1%)	
N1-3	94 (67.6%)	123 (60.9%)	
**TNM stage^†^**			0.239
III	49 (35.2%)	84 (41.6%)	
IV	90 (64.8%)	118 (58.4%)	
**Site**			0.277
laryngeal	66 (47.5%)	108 (53.5%)	
hypopharyngeal	73 (52.5%)	94 (46.5%)	
**Surgical margin**			0.681
positive	10 (7.2%)	17 (8.4%)	
negative	129 (92.8%)	185 (91.6%)	
**Vascular invasion**			0.461
yes	13 (9.4%)	24 (11.9%)	
no	126 (90.6%)	178 (88.1%)	
**Adjuvant radiotherapy**			0.781
yes	101 (72.7%)	144 (71.3%)	
no	38 (27.3%)	58 (28.7%)	
**Adjuvant chemotherapy**			0.094
yes	54 (38.8%)	97 (48.1%)	
no	85 (61.2%)	105 (51.9%)	
**Smoking history**			0.602
yes	114 (82.0%)	170 (84.2%)	
no	25 (18.0%)	32 (15.8%)	
**Drinking history**			0.477
yes	62 (44.6%)	98 (48.5%)	
no	77 (55.4%)	104 (51.5%)	

Abbreviation: AAPR, albumin-to-alkaline phosphatase ratio; TNM, tumor-node-metastasis; ALB, albumin; ALP, alkaline phosphatase; †Tumor-node-metastasis staging system was proposed by the 7^th^ edition American Joint Committee on Cancer (AJCC).

**Table 2:** Survival analysis

Group	Low level of AAPR (N=139)		High level of AAPR (N=202)		P value
	3-year	5-year	3-year	5-year	
DFS	58.6%	46.0%	81.2%	71.9%	0.000
OS	70.8%	69.0%	84.6%	72.6%	0.000
LRFS	63.4%	55.7%	85.2%	78.6%	0.000
DMFS	88.1%	77.8%	90.6%	83.7%	0.140

Abbreviations: OS, overall survival; DFS, disease-free survival; LRFS, local recurrence-free survival; DMFS, distant metastasis-free survival. OS, DFS, LRFS and DMFS were calculated by Kaplan-Meier method; P value was calculated by Log-Rank test.

**Table 3:** Univariate analysis of DFS and OS

Variable	DFS		OS	
	HR (95%CI)	*P* value	HR (95%CI)	*P* value
**Age, year**				
<65 vs ≥65	1.071 (0.720-1.594)	0.734	0.648 (0.449-0.934)	0.020
**Sex**				
Male vs female	0.449 (0.111-1.817)	0.262	0.451 (0.112-1.825)	0.265
**AAPR level**				
Low vs high	2.370 (1.667-3.378)	0.000	2.049 (1.443-2.901)	0.000
**T stage^†^**				
T1-2 vs T3-4	0.812 (0.487-1.354)	0.425	0.855 (0.513-1.426)	0.548
**N status^†^**				
N0 vs N1-3	0.568(0.386-0.836)	0.004	0.658 (0.452-0.956)	0.028
**Surgical margin**				
Positive vs negative	1.108(0.562-2.183)	0.767	1.045 (0.547-1.993)	0.895
**Vascular invasion**				
Yes vs no	2.114(1.311-3.413)	0.002	1.340 (0.769-2.336)	0.302
**Adjuvant radiotherapy**				
Yes vs no	0.824(0.552-1.230)	0.344	1.051 (0.717-1.540)	0.801
**Adjuvant chemotherapy**				
Yes vs no	0.761(0.537-1.078)	0.125	0.786 (0.555-1.112)	0.174
**Smoking history**				
Yes vs no	1.100(0.694-1.741)	0.686	0.753 (0.446-1.271)	0.288
**Drinking history**				
Yes vs no	0.894(0.631-1.266)	0.527	0.998 (0.705-1.413)	0.991

Abbreviations: AAPR, albumin-to-alkaline phosphatase ratio; DFS, disease-free survival; OS, overall survival. †Tumor-node-metastasis staging system was proposed by the 7^th^ edition American Joint Committee on Cancer (AJCC).

**Table 4:** Multivariate analysis of DFS and OS

Variable	DFS		OS	
	HR (95%CI)	*P* value	HR (95%CI)	*P* value
**AAPR**				
low vs high	2.457 (1.718-3.509)	0.000	2.016 (1.418-2.865)	0.000
**N status**				
N0 vs N1-3	0.637 (0.431-0.942)	0.024	0.632 (0.432-0.925)	0.018
**Age, year**				
<65 vs ≥65	NA		0.607 (0.418-0.881)	0.009
**Vascular invasion**				
Yes vs no	2.193 (1.348-3.571)	0.002	NA	

Abbreviations: DFS, disease-free survival; OS, overall survival; CI, confidence interval; HR, hazard ratio.
